# Locus-specific hypomethylation of the mouse IAP retrotransposon is associated with transcription factor-binding sites

**DOI:** 10.1186/s13100-017-0105-0

**Published:** 2017-12-13

**Authors:** Ken-ichi Shimosuga, Kei Fukuda, Hiroyuki Sasaki, Kenji Ichiyanagi

**Affiliations:** 1 Division of Epigenomics and Development, Medical Institute of Bioregulation, and Epigenome Network Research Center, Kyushu University, 3-1-1 Maidashi, Higashi-ku, Fukuoka, 812-8582 Japan; 2 Trygroup Incorporated, 1-8-10 Kudankita, Chiyoda-ku, Tokyo, 102-0073 Japan; 3 Cellular Memory Laboratory, RIKEN, Wako, Saitama, 351-0198 Japan; 4 Laboratory of Genome and Epigenome Dynamics, Department of Applied Molecular Biosciences, Graduate School of Bioagricultural Sciences, Nagoya University, Nagoya, 464-8601 Japan

**Keywords:** DNA methylome, Intracisternal A particle, Endogenouse retrovirus, Transcription factor, Spermatogenesis, Mouse

## Abstract

**Background:**

Intracisternal A particle (IAP) is one of the most transpositionally active retrotransposons in the mouse genome, but its expression varies between cell types. This variation is believed to arise from differences in the epigenetic state (e.g., DNA methylation) of the 5′ long terminal repeat (LTR), where transcription starts. However, owing to the high copy number and high sequence similarity between copies, it is difficult to analyze the epigenetic states of individual IAP LTRs in a comprehensive manner.

**Results:**

We have developed a method called Target Enrichment after Post-Bisulfite Adaptor Tagging (TEPBAT) to analyze the DNA methylation states of a large number of individual retrotransposon copies at once. Using this method, we determined the DNA methylation levels of >8500 copies of genomic IAP LTRs (almost all copies that we aimed to target by the PCR primers) in the sperm and tail. This revealed that the vast majority of the LTRs were heavily methylated in both sperm and tail; however, hypomethylated copies were more frequently found in the sperm than in the tail. Interestingly, most of these hypomethylated LTRs were solo-type, belonged to specific IAP subfamilies, and carried binding sites for transcription factors (TFs) that are active in male germ cells.

**Conclusions:**

The current study revealed subfamily- and locus-specific hypomethylation of IAP LTRs, and suggests that binding of TFs is involved in the protection from DNA methylation, whereas the IAP internal sequence enhances methylation. Furthermore, the study demonstrated that TEPBAT offers a cost-effective method for a variety of DNA methylome studies that focus on retrotransposon sequences.

**Electronic supplementary material:**

The online version of this article (10.1186/s13100-017-0105-0) contains supplementary material, which is available to authorized users.

## Background

Approximately 40% of the mammalian genome comprises several million copies of retrotransposons [1], which include long terminal repeat (LTR) retrotransposons, long interspersed elements, and short interspersed elements. These retrotransposons are amplified by retrotransposition, a process in which their transcribed RNA is utilized to make a DNA copy by reverse transcription. It has been reported that retrotransposition causes heritable diseases, such as hemophilia A and B, muscular dystrophy, and X-linked agammaglobulinemia in humans [2]. In mice, 10–12% of spontaneous mutant alleles arise as a result of the retrotransposition of LTR retrotransposons, such as intracisternal A particle (IAP), early transposon (ETn), and MusD [3]. To diminish the transpositional activity, retrotransposon expression can be epigenetically regulated at the transcriptional level by DNA methylation at CpG sites and by repressive histone modifications. A knockout mutation of the mouse *Dnmt1* gene, which encodes a maintenance-type DNA methyltransferase, has been shown to cause derepression of IAP in whole embryos because of a passive loss of DNA methylation [4]. Likewise, several LTR retrotransposons are derepressed in mouse embryonic stem cells having deletion of the *Setdb1* gene, which encodes a protein methyltransferase acting on the lysine-9 residue of histone H3 (H3K9). The derepression is attributable to the loss of H3K9 trimethylation at the LTRs [5, 6].

The mouse genome contains approximately 4000 copies of full-length or nearly full-length IAP, which consist of two LTRs at both ends and an internal sequence carrying *gag*, *pro*, and *pol* genes. In addition, the genome contains approximately 5000 copies of solo LTRs of IAP, where an LTR alone is present. The sequence of IAP LTR is 300- to 450-bp long, and contains 15–25 CpG sites. In somatic cells, the vast majority of these CpG sites are heavily methylated, and consequently, IAP expression level is very low [4]. However, it has been reported that a small fraction of LTRs escape methylation with significant variation between individuals; sometimes they behave as metastable epialleles (i.e., LTR copies in the *A* ^*vy*^ *, A* ^*iap*^, and *Axin* ^*fu*^ loci) [7, 8]. Conversely, higher IAP expression is detected in preimplantation embryos (from the 8-cell to blastocyst stages) [9] and in prospermatogonia and spermatogonia [10], which are precursors of spermatozoa. Although DNA methylation levels in IAP copies are indeed low in blastocysts, those in spermatogonia are high [11–13]. Therefore, it is possible that in spermatogonia, some specific IAP copies are hypomethylated to serve as a source of IAP expression, whereas the vast majority remains highly methylated. It would be therefore interesting to analyze the methylation levels in individual IAP copies in a genome-wide manner, rather than to analyze them in bulk (via PCR using primers for the IAP consensus sequence). The development of the whole-genome bisulfite shotgun sequencing method offers an opportunity to analyze DNA methylation levels in individual IAP copies (i.e., the IAP methylome). However, the cost to obtain sufficient sequence depth is high. To reduce the cost while maintaining the comprehensive depth that is required, a method called high-throughput targeted repeat element bisulfite sequencing (HT-TREBS) [14] was recently developed. The study confirmed the high level of DNA methylation at vast majority of IAP LTRs, while a small subset are hypomethylated. However, the mechanism underlying their hypomethylation remains unknown.

In the present study, we determined the IAP methylomes in germ and somatic cells using a method where IAPs and their flanking sequences were selectively amplified using a random primer and an IAP-specific primer after bisulfite treatment of genomic DNA. Deep sequencing analysis revealed that specific IAP subfamilies were hypomethylated, whereas a vast majority were highly methylated. Most of the hypomethylated copies were solo LTRs and carried binding motifs for specific transcription factors (TFs). We discuss a possible role of TFs in protecting these copies from methylation and a role for the internal sequence in recruiting methylation enzymes.

## Results

### Determination of DNA methylation levels of IAP LTR copies by the TEPBAT method

**Fig. 1:**
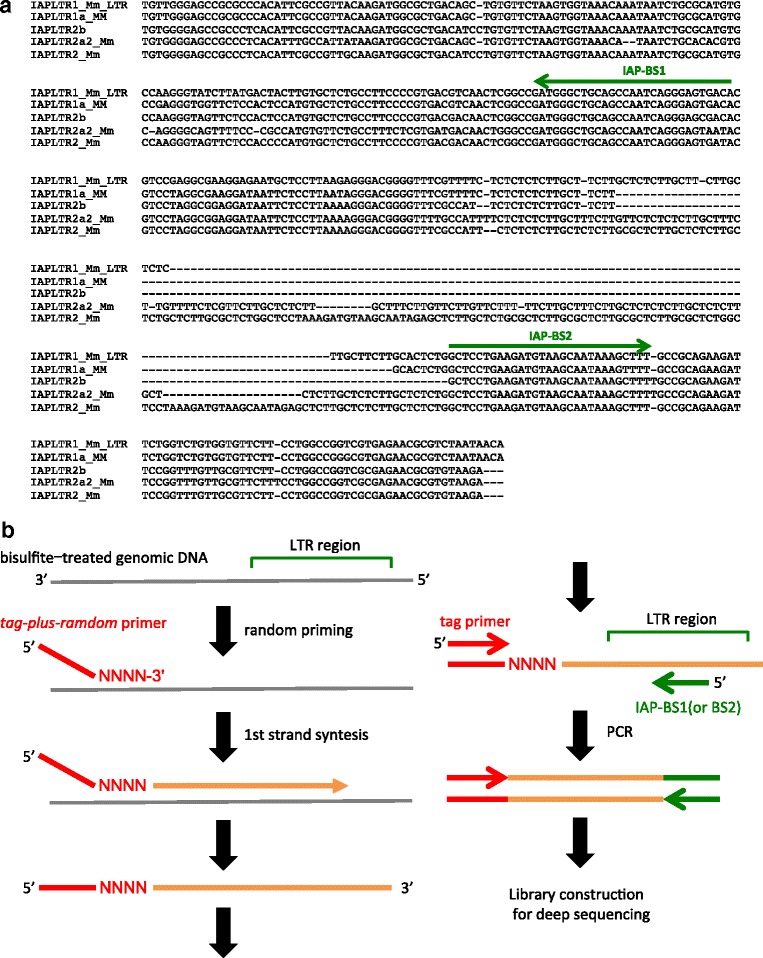
Experimental design of TEPBAT. **a** Nucleotide sequence alignment of the consensus sequences of IAPLTR1, IAPLTR1a, IAPLTR2, IAPLTR2a2, and IAPLTR2b. The primer regions and orientations (IAP-BS1 and IAP-BS2) used in TEPBAT are indicated as green arrows. **b** Experimental design to enrich IAP regions in the bisulfite-treated genomic DNA. After bisulfite treatment, the first DNA strand was synthesized using the *tag-plus-random* primer, and the IAP regions were amplified by PCR using the specific primer (green arrow) and tag primer (red arrow)

To selectively obtain bisulfite sequencing data for the IAP LTR sequences, we developed a method that was modified from the PBAT library preparation method designed for whole-genome bisulfite sequencing [15]. In our method (Fig. 1a), designated as TEPBAT (Target Enrichment after Post-Bisulfite Adaptor Tagging), the first DNA strand was synthesized using bisulfite-treated DNA as a template and the *tag-plus-random* primer, which consisted of a random tetramer and a specific tag sequence. The random tetramer end of the primer enabled genome-wide synthesis of the first DNA strand. In the second step, IAP-containing regions were selectively amplified by PCR with the IAP-specific primer and tag-sequence primer (which was almost same as the *tag-plus-random* primer, but did not contain the random tetramer sequence).

**Fig. 2:**
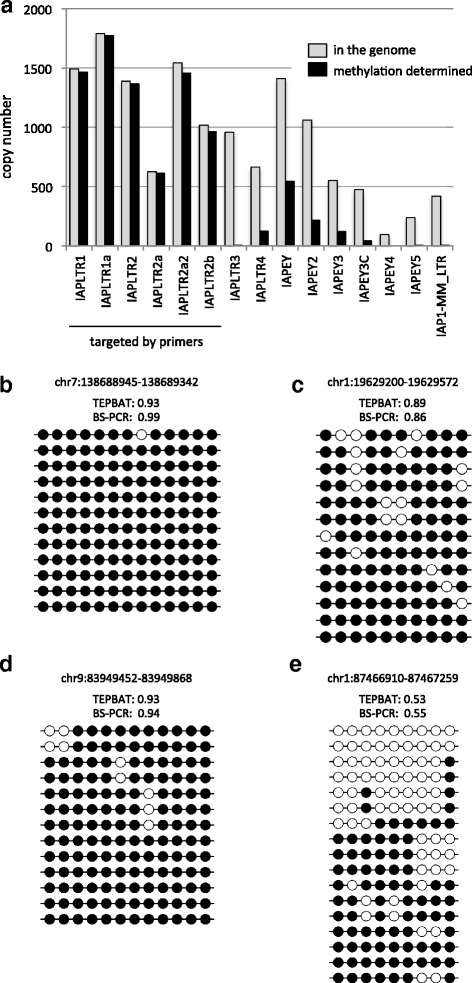
DNA methylation levels of individual IAP LTR copies. **a** The number of LTR copies of the subfamilies. The numbers of the total genomic copies are shown in gray and those for which methylation levels were analyzed (total CpG methylation calls of ≧20) are shown in black. **b**-**e** Bisulfite-PCR results for selected loci in the tail DNA. Closed and open circles indicate methylated and unmethylated CpG sites, respectively. Each row represents a clone of bisulfite-PCR products. The genomic location (mm10) of the loci and their methylation levels (expressed as a fractional value) determined by TEPBAT and bisulfite-PCR (BS-PCR) are shown

Genomic copies of the IAPLTR1_Mm_LTR (referred to as IAPLTR1), IAPLTR1a_MM (IAPLTR1a), IAPLTR2_Mm (IAPLTR2), IAPLTR2a2_Mm (IAPLTR2a2), and IAPLTR2b subfamilies comprise more than half of the total IAP LTRs in the genome (see Fig. 2a) and are less diverged because of recent retrotransposition. Therefore, in the present study, we targeted these copies for methylation analysis. To selectively amplify these copies, the IAP-BS1 and IAP-BS2 primers were designed in regions that are highly conserved among the subfamilies (Fig. 1b). IAP-BS1 was designed in the reverse orientation to amplify the 5′ region and the upstream flanking sequence, whereas IAP-BS2 was designed in the forward orientation to amplify the 3′ region and the downstream flanking sequence. We note that the IAP-BS2 primer could hybridize to IAPEY_LTR (IAPEY) and IAPEY2_LTR (IAPEY2) weakly. The PCR products were ligated to the sequencing adaptor, and paired-end deep sequencing was performed on HiSeq2500 so that one of the paired reads facilitated mapping uniquely to the genome, whereas the other contained the IAP sequence.

To investigate and compare the IAP methylation profiles of germ and somatic cells, we prepared genomic DNAs from the sperm and tail of the same male mouse (the C57BL6/J strain). Using these DNAs, we obtained 86 and 81 million sequencing read pairs, respectively. After removing the primer sequences (including the region of the random tetramer) and low-quality nucleotides, the read pairs were mapped to the mouse genome sequence to call the methylation state of each CpG site (see Methods). On average, about 10 CpG sites in an LTR were covered by the reads with an average sequencing depth of about 320 (yielding about 320 × 10 = 3200 methylation calls). To calculate the methylation level at individual IAP LTRs (expressed as a fractional value [0.00–1.00] and calculated by dividing the methylated cytosine calls by a sum of methylated and unmethylated cytosine calls), the methylation levels at CpG sites within an LTR were averaged. LTRs with <20 CpG methylation calls were excluded from the analysis, retaining the methylation data of 8698 and 8517 LTRs for the sperm and tail, respectively. For comparative analysis, we focused on 8153 LTRs whose methylation levels could be analyzed in both samples. These LTRs included >97% of genomic copies of the targeted IAP subfamilies (IAPLTR1/1a/2/2a/2a2/2b), and 40 and 20% of genomic IAPEY and IAPEY2 LTRs, respectively (Fig. 2a). To validate the acquired methylation data, we determined the methylation levels of selected LTR loci by bisulfite-PCR using the tail DNA. This data was consistent with the TEPBAT data (Fig. 2b-e).

**Fig. 3:**
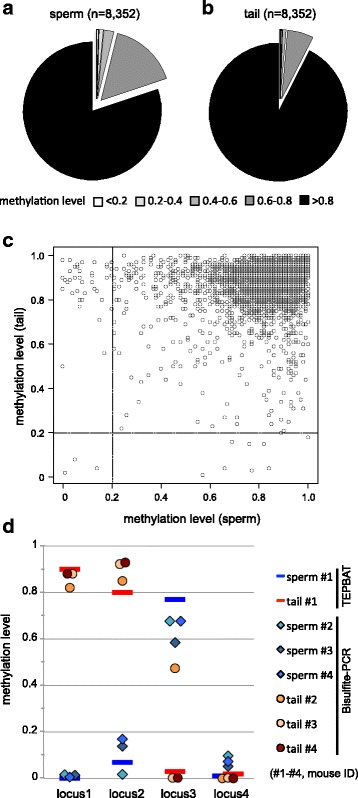
IAP LTR methylation landscape. **a**, **b** Pie charts of LTR copies categorized by their methylation levels in the sperm (**a**) and in the tail (**b**). Methylation levels are indicated at the bottom. **c** Scatter plot of DNA methylation levels of IAP LTR copies (*n* = 8153) in the sperm and tail (x- and y-axis, respectively). **d** Bisulfite-PCR results for selected loci in the sperm and tail in different individuals (mouse ID #2, #3, and #4). These individuals are different from the one (mouse ID #1) used for TEPBAT analysis. Brue (sperm) and red (red) bars indicate the methylation levels determined by TEPBAT. Blue (sperm) and red (tail) circles indicate the methylation levels of three individuals determined by sequencing of 10 to 16 PCR clones. The genomic location (mm10) of the loci are as follows: locus1, chr8:42,217,148-42,217,495 (IAPEY); locus2, chr18:87,502,570-87,503,025 (IAPLTR2a2); locus3, chr13:4,942,652-4,943,122 (IAPLTR2); locus4, chr3:96,489,247-96,489,584 (IAPLTR1)

High-throughput analysis revealed that 6677 (80% of the total LTRs analyzed) and 7726 (93%) LTRs exhibited methylation levels of >0.8 in the sperm and tail, respectively (Fig. 3a, b). This is consistent with the notion that IAP LTRs are generally methylated. However, a small fraction of LTRs showed medium (0.2–0.8) and low (≦0.2) methylation levels. Hypomethylation (methylation level ≦0.2) was tissue-specific, and only three LTR copies were hypomethylated in both the sperm and tail (Fig. 3c). In the sperm, 43 and 1612 LTR copies showed low and medium methylation levels, respectively (Fig. 3a), whereas 14 and 612 copies showed low and medium methylation levels in the tail, respectively (Fig. 3b). Therefore, although generally hypermethylated, the IAP LTR methylation level is relatively low in germ cells as compared to somatic cells.

The IAP LTR methylation levels could vary between individuals. Therefore, different individuals were analyzed for several LTR loci in the tail and sperm by bisulfite-PCR (Fig. 3d). The methylation levels at locus3 (IAPLTR2) in tail showed significant variation between individuals (0.47 in individual #2, whereas 0.03 in individual #1 and <0.01 in individuals #3 and #4). However, the methylation levels at other loci were largely conserved among the four individuals analyzed.

### Solo LTRs of specific subfamilies display hypomethylation

**Fig. 4:**
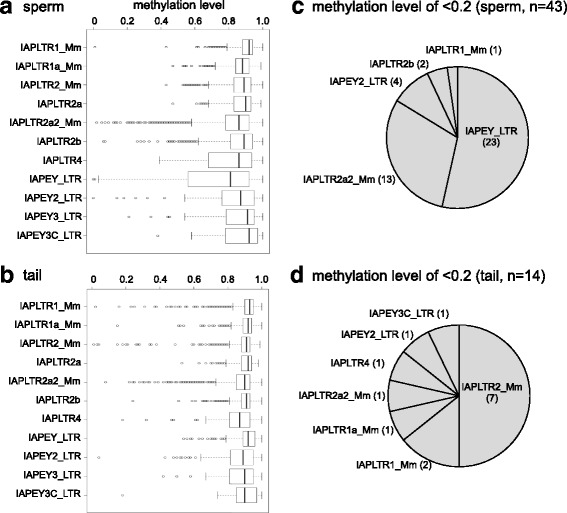
Specific subfamilies are enriched in hypomethylated copies. **a**, **b** Box plots of DNA methylation levels of IAP subfamilies in the sperm (**a**) and in the tail (**b**). **c**, **d** Subfamily distributions of hypomethylated copies (methylation level < 0.2) in the sperm (**c**) and in the tail (**d**) are shown in pie charts. The numbers in parentheses indicate actual copy numbers

We investigated whether each subfamily showed specific methylation profiles. The median methylation level was >0.8 for all subfamilies (Fig. 4a, b); however, we found that IAPEY and IAPLTR2a2 in the sperm and IAPLTR2 in the tail contained some hypomethylated loci. Indeed, out of the 43 hypomethylated LTRs in the sperm (methylation level of ≦0.2), 23 (53%), and 13 (30%) belonged to IAPEY and IAPLTR2a2, respectively (Fig. 4c). In the tail, 7 (50%) out of the 14 hypomethylated LTRs belonged to IAPLTR2 (Fig. 4d). Therefore, even though most subfamilies showed high methylation levels, a fraction of copies of the specific subfamilies were hypomethylated. IAPEY is a relatively young subfamily accumulated in the Y chromosome [16] with 60% of ~1400 genomic copies being in the Y chromosome (Additional file 1: Figure S1). Because the fraction of analyzed IAPEY copies was small (Fig. 2a), it should be noted that the number of hypomethylated IAPEY copies may be underestimated. The Y-chromosome copies are more divergent than those in autosomes (Additional file 1: Figure S1), and most (75%) of the copies of which methylation levels were determined reside in autosomes. Consequently, almost all of the copies showing hypomethylation in sperm are also autosomal.

**Fig. 5:**
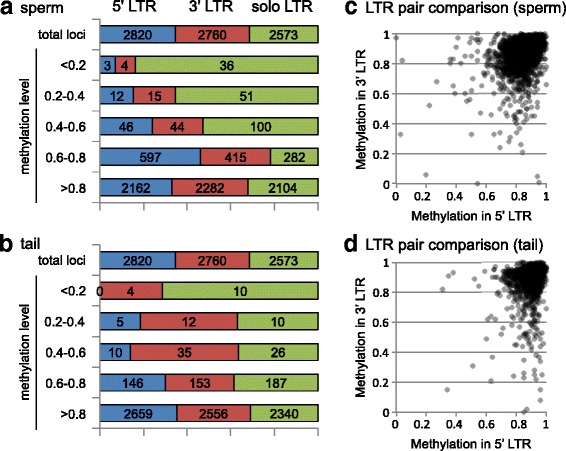
LTR features and DNA methylation. The LTR copies (*n* = 8153) are categorized into 5′ LTR (blue), 3′ LTR (red) and solo LTR (green) and into five groups according to their methylation levels in the sperm (**a**) and in the tail (**b**). The numbers indicate actual copy numbers. (**c**, **d**) Comparison of methylation levels in sperm (**c**) and tail (**d**) in the two LTRs within the same elements. For IAP copies having both 5′ and 3′ LTRs, the methylation levels in the 5′ LTRs (x axis) are plotted against those in the 3′ LTRs (y axis)

Even in these subfamilies, most loci were heavily methylated. To elucidate the mechanism of hypomethylation, we analyzed the sequence features of the hypomethylated loci. First, because LTR sequences exist as a terminal repeat or as a solo LTR, we determined whether these features affect methylation levels. We manually annotated all IAP LTRs in the genome as 5′ LTR, 3′ LTR, or solo LTR (Additional file 2: Table S1). The 8153 LTRs, for which methylation levels were determined in both tissues, included comparable numbers of 5′ LTRs, 3′ LTRs, and solo LTRs (2820, 2760, and 2573 copies, respectively). However, solo LTRs were significantly enriched with hypomethylated loci; for example, 36 (84%) and 10 (71%) of hypomethylated loci in the sperm and tail, respectively, were solo LTRs (Fig. 5a, b). On the other hand, 5′ and 3′ LTRs were less frequently hypomethylated. For these hypomethylated copies, it was generally observed in both sperm and tail that only one LTR was hypomethylated whereas the other in the same element was hypermethylated (Fig. 5c, d), suggesting that methylation levels of the 5′ and 3′ LTRs in an element are regulated independently of each other.

It should be noted that even for solo LTRs of the specific subfamily, not all loci were hypomethylated. Therefore, for these solo LTRs, we investigated whether some genomic and epigenomic features were associated with hypomethylation. However, we did not find a strong correlation while analyzing their locations relative to genes (i.e., promoter, exon, intron, and intergenic), the distance to the nearest transcriptional start site, GC content of their neighboring regions (in 1-, 10-, or 100-kb bins), the number of CpG sites in their neighboring regions (in 1-, 10-, or 100-kb bins), the nucleotide divergence compared to the respective consensus sequences, methylation levels of their flanking regions in sperm [17], or their methylation levels in primordial germ cells at embryonic day 13.5 [18] (data not shown).

### Hypomethylated loci have specific sequences

**Fig. 6:**
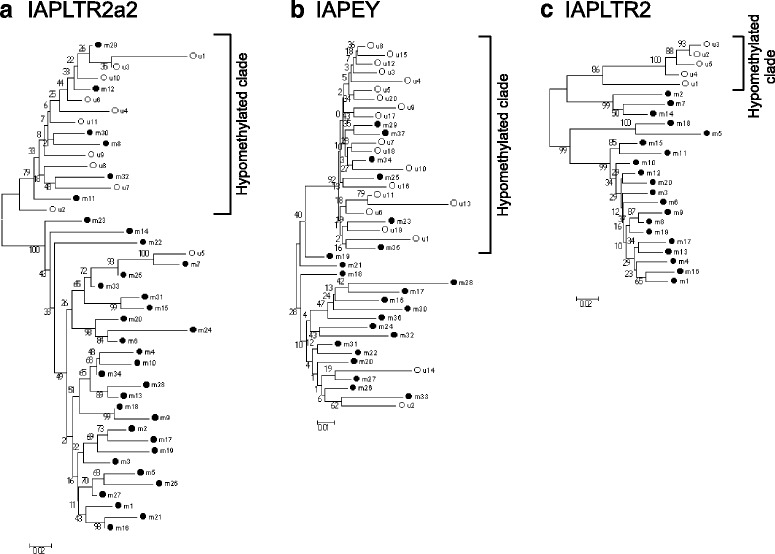
Clustering of hypermethylated LTR sequences in the phylogenetic trees. Neighbor-joining trees of members of (**a**) IAPLTR2a2, (**b**) IAPEY, and (**c**) IAPLTR2. Nucleotide sequences of the hypomethylated copies and randomly selected hypermethylated copies of the same subfamily were aligned using Clustal X [37] to generate a neighbor-joining tree on Mega5 [19]. Open and closed circles indicate hypomethylated and hypermethyated copies, respectively. In each panel, u1, u2, u3, etc. are locus names of hypomethylated copies, and m1, m2, m3, etc. are locus names of hypermethylated copies. The clade that includes the most hypomethylated copies (designated as hypomethylated clade) is indicated. The numbers on the nodes indicate bootstrap values (1000 replicates)

The sequences of LTR copies even of the same subfamily display slight variations. We analyzed the LTR sequences to see whether specific sequences are associated with hypomethylation. We aligned the sequences of the 11 IAPLTR2a2 sequences determined to be hypomethylated in the sperm, with randomly selected 34 hypermethylated (methylation levels of about 0.92) IAPLTR2a2 sequences. Clustering of the sequences by the neighbor-joining method using Mega5 [19] revealed that these 45 sequences were divided into two major clades (Fig. 6a), one of which contained most of the hypomethylated LTRs. These were designated as the hypomethylated clade. Likewise, the 20 hypomethylated (in the sperm) and 22 randomly selected hypermethylated IAPEY sequences were divided into two clades, where the hypomethylated sequences were clustered together (Fig. 6b). The five hypomethylated (in the tail) and 20 randomly selected hypermethylated IAPLTR2 sequences were divided into two clades; the hypomethylated sequences were again clustered together (Fig. 6c).

**Fig. 7:**
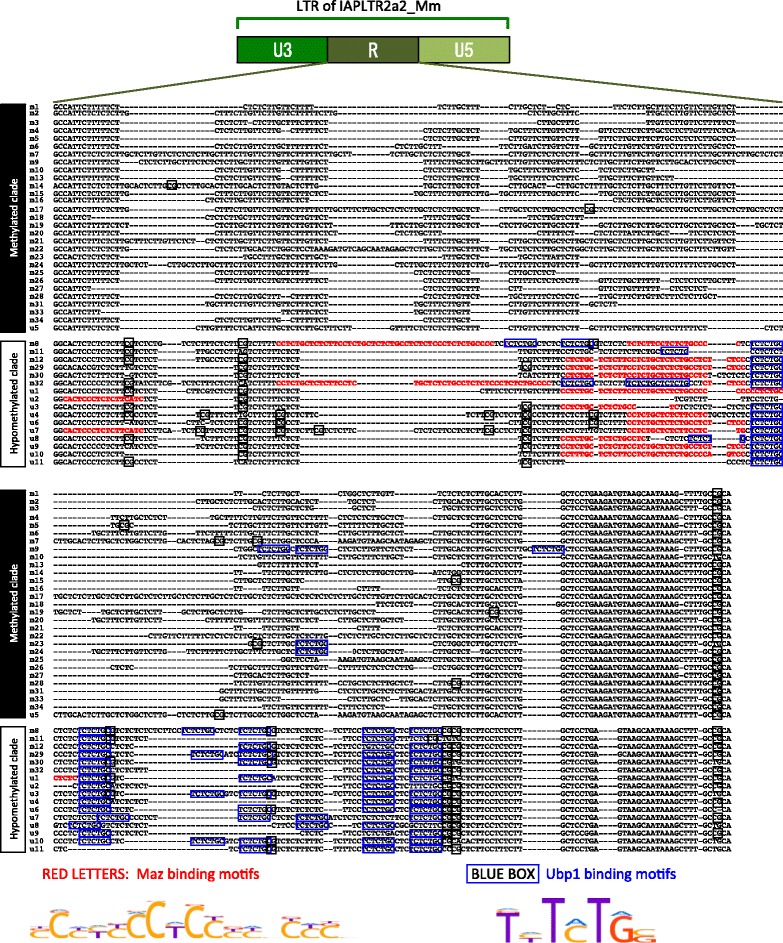
Hypomethylated clade of IAPLTR2a2 is characterized by the presence of Maz- and Ubp1-binding motifs. The nucleotide sequences of the R region of the LTR (see top for a schematic view of the LTR sub-regions) of IAPLTR2a2 copies belonging to the methylated and hypomethylated clades were aligned by Clustal X [37]. Maz-binding motifs are highlighted in red. Ubp1-binding sites are indicated by a blue box. CpG sites are indicated by a black box. Members of the hypomethylated clade carry multiple Maz and Ubp1 sites, whereas almost all members of the hypermethylated clade carry none. Sequence Logo representation of the binding motifs is shown at the bottom

In each case, the clear clustering (bootstrap values >86) suggested the presence of single nucleotide variations (SNVs) or sequence blocks that discriminate the two clades. Indeed, we identified several nucleotide positions that were conserved in the hypomethylated clade but not in the other clade. Given the possibility that sequence differences may result in differences in binding of TFs, we searched sequence motifs for TF binding by FIMO [20]. This revealed that eight TF-binding motifs (Prop1, Spi1, Ubp1, Hnf4g, Mitf, Maz, Mafk, and Nf2l2) were significantly enriched in the hypomethylated clade of IAPLTR2a2. In particular, the motifs for Maz and Ubp1 were absent in all (Maz) or most (Ubp1) of hypermethylated sequences, whereas all hypomethylated sequences carried multiple motif sequences. These hypomethylated clade-specific Ubp1 and Maz-binding motifs were all located in the R region of the LTR (Fig. 7), which is known to play a role in transcriptional regulation and shows extensive sequence variation [21]. It is noteworthy that out of the eight TFs mentioned above, Maz and Ubp1 are expressed in spermatogenic cells in the published transcriptome data [22]. Therefore, it is conceivable that the binding of these TFs during spermatogenesis reduces the methylation level of their binding sites in these cells and spermatozoa. Interestingly, the hypomethylated sequences tend to have more CpG sites than hypermethylated sites, especially around the TF binding motifs (Fig. 7).

For the IAPEY subfamily, we also identified clade-specific SNVs, but could not identify clade-specific TF-binding motifs. In contrast, for the IAPLTR2 subfamily, we identified Plag1 and Sp1 binding motifs that were specific to the hypomethylated clade. The expression of these TFs in the adult tail is unknown.

## Discussion

In the present study, we developed a method called TEPBAT to effectively analyze DNA methylation levels of genomic copies of a specific retrotransposon in bulk. In this method, a single lane of a HiSeq run was sufficient to obtain methylation data of thousands of genomic IAP LTR copies. It seems conceivable that other interspersed repeats can also be analyzed by TEPBAT if appropriately designed primers are used.

**Fig. 8:**
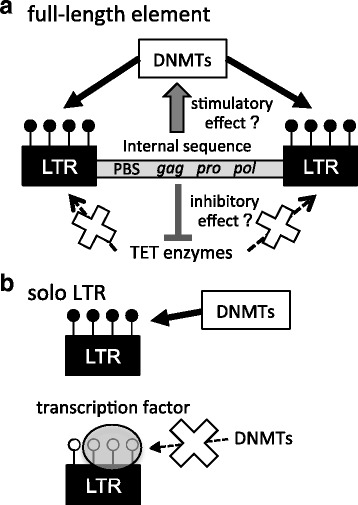
Models for molecular mechanisms that regulate IAP methylation. **a** In copies containing the internal sequence, some parts of the internal sequence may stimulate DNA methylation in their flanking LTR sequences. Closed circles represent methylated CpG sites in LTRs. DNMTs, DNA methyltransferases. TET enzymes, Ten-Eleven translocation enzymes (catalyzing oxidation of methylcytosine). PBS, primer binding site. **b** Most solo LTR copies are methylated by DNMTs (top). However, copies carrying TFBSs bind to these TFs if the TFs are expressed in the cell. This binding inhibits the action of DNMTs (bottom). The bound transcription factor is represented as a gray oval. Open circles represent unmethylated CpG sites in the LTRs

Although most of the genomic IAP LTR copies were heavily methylated in the sperm and tail, we found that LTRs of a specific subfamily exhibited a tendency to be hypomethylated, especially when specific sequences were present. This implies that the epigenetic state of IAP is dictated by the genetic sequence. Indeed, we found that several TF-binding motifs are specifically associated with hypomethylated sequences, which suggests that TF binding interferes with DNA methyltransferases, and subsequent methylation modifications (Fig. 8). A similar association between variation of TF-binding motifs and methylation levels has been observed for unique sequences as well [23–26]. However, not all copies in the hypomethylated clade were hypomethylated, suggesting that additional factor(s) may also be involved in the regulation of IAP methylation.

In contrast to solo LTRs, hypomethylation of 5′ and 3′ LTRs was very infrequent. A possible explanation would be that the internal sequence of IAP (*gag*, *pro*, *pol*, and 5′ and 3′ untranslated regions) carry a sequence(s) bound to a protein(s) important for de novo and/or maintenance of DNA methylation. This type of methylation regulation may include Krüppel-associated box containing zinc finger proteins (KRAB-ZFPs) that regulate the epigenetic state of their binding sequences. KRAB-ZFPs can bind DNA in a sequence-specific manner via the ZF domain, while they can also bind the KAP1 protein via the KRAB domain [27, 28]. The KAP1 protein in turn binds SetDB1, histone deacetylases, and DNA methyltransferases; therefore, the KRAB-ZFP/KAP1 complex induces a repressive chromatin state in the KRAB-ZFP binding regions. For example, among several hundred mouse KRAB-ZFPs, ZFP809 binds an endogenous copy of Mouse Leukemia Virus to induce DNA and histone H3K9 methylation in the internal sequence and LTRs [29]. Likewise, the human KRAB-ZFPs, ZNF91 and ZNF93, bind to SVA and L1 retrotransposons, respectively, leading to an induction of histone H3K9 methylation [30]. It is possible that a certain KRAB-ZFP binds to the IAP internal sequence to induce DNA methylation. ZFP819 may be a candidate because the protein has been shown to bind the IAP internal sequence (5’ UTR and the *pol* region of IAPEZ-int) and LTR (IAPLTR1a), and to regulate IAP expression [31]. In addition, not mutually exclusive, it is also possible that the internal sequence inhibits binding of the TET1, TET2, and/or TET3 enzymes, which catalyze oxidation of methylcytosine leading to loss of methylation [32].

It has been proposed that some genomic copies of retrotransposons can serve as a source of epigenetic and phenotypic diversity within a population [33]. IAP is of particular interest in this regard because several IAP copies have been shown to behave as metastable epialleles [7, 8]. These copies in the *A* ^*vy*^ *, A* ^*iap*^, and *Axin* ^*fu*^ alleles belong to the IAPLTR1 subfamily and not present in our mouse strain (C57BL6/J) [7, 8]. Thus, the mechanism for their occasional hypomethylation remains unknown. To understand the retrotransposon-mediated heritable epigenetic changes more profoundly, genome-wide analyses of epigenetic states of these sequences must be performed for different tissues of many individuals with the discrimination of individual copies. The results here demonstrate that TEPBAT offers a cost-effective method for such studies.

## Conclusions

Using TEPBAT, we revealed that solo LTRs of specific subfamilies (IAPLTR2a2 and IAPEY in sperm and IAPLTR2 in tail) tend to be hypomethylated. Binding of TFs seems to account for hypomethylation of these copies, emphasizing the importance of TFs in regulating the epigenome. It should be noted, however, that the hypomethylation of IAPLTR2a2 and IAPEY copies in the sperm does not explain the higher expression of IAP in spermatogenic cells because most of the hypomethylated copies were solo LTRs lacking the internal sequence. Although further studies will be required to solve the issue, our method can be applied to a variety of DNA methylome studies that focus on retrotransposon sequences.

## Methods

### Sequence library construction and paired-end sequencing

High-molecular-weight genomic DNA was prepared from tail and sperm samples of the same adult mouse (C57BL6/J) by a standard procedure. The DNA was treated with 10 M bisulfite (sodium and ammonium salt), as described previously [34]. The resultant DNA (25 ng) was incubated with 80 nM *tag-plus-random* primer (5’-GCAGTGAACTGACTACAGGNNNN-3′) in Klenow buffer (10 mM TrisHCl, 10 mM MgCl_2_, 1 mM DTT, and 125 μM each of dNTPs) at 94 °C for 5 min, then quickly cooled down to 4 °C. After 7.5 U of Klenow Fragment exo^−^ (New England Biolab) was added, the reaction mixture was incubated at 4 °C for 15 min. The temperature was then increased to 37 °C at the rate of 1 °C per minute and maintained at 37 °C for 90 min. After heat inactivation at 70 °C for 10 min, DNA was purified using AMPure XP (Beckman Coulter). IAP-containing genomic regions were amplified by PCR (25 cycles of 94 °C for 15 s, 55 °C for 30 s, and 68 °C for 30 s) using KOD-plus neo (Toyobo), tag primer (5’-CAGTGAAC- TGACTACAGG-3′), and either of IAP-BS1 (5’-GGGGAAGGTAGAGTATAWG-3′) or IAP-BS2 (5′-GGTTTTTGAAGATGTAAGTAATAAAGTTTT-3′) primers. The 350–450-bp long PCR products were purified by electrophoresis using a 2% agarose gel, and Illumina sequencing adaptors were added to their ends using the TruSeq DNA Sample Prep kit (Illumina). Paired-end 100-bp sequencing was carried out on a HiSeq2500 in the highoutput mode (one lane per tissue sample with equal amounts of the IAP-BS1 and IAP-BS2 libraries being mixed). About 80% of read pairs contained the tag and IAP-BS1/BS2 sequences. After removing the primer sequences (including the random tetramer region) and low-quality bases by a perl script, the read pairs were mapped to the genome (mm10) by Bismark [35] with default parameters to call the methylation state at each CpG position. With this set of parameters, read pairs with multiple hits were discarded. The mapping efficiency was 22–25%. The low mapping efficiency in comparison to PBAT (typically 50–70%) is likely due to the fact that many LTR copies are flanked by repeat elements such as IAP internal sequences.

### Bisulfite-PCR analysis

Using the bisulfite-treated tail DNA, touch-down PCR was performed as described previously [34] with locus-specific primers (forward and reverse primers were designed for the LTR and flanking region, respectively). PCR fragments were cloned into the pGEM-Teasy vector (Promega) and sequenced using 3730 DNA Analyzer (Applied Biosystems).

### Methylation analysis of IAP LTR sequences

The genomic positions of IAP sequences were obtained from the repeatmasker table from the UCSC genome browser [36]. For each LTR copy, their features (5′ LTR, 3′ LTR, or solo LTR) were manually determined (Table S1). To calculate LTR methylation levels, the methylation levels of CpG sites within the LTRs were averaged. Only those LTRs having ≧20 CpG methylation calls (sum of sequencing depth at CpG sites) were used for further analysis. Only LTRs present in the reference sequence were analyzed.

## Additional files

Additional file 1: Sequence divergence of IAPEY_LTR genomic copies. (PDF 106 kb)

Additional file 2: Table S1. Annotation and methylation levels of each IAP fragment. (XLSX 2426 kb)

## Abbreviations

H3K9: The lysine-9 residue of histone H3
IAP: Intracisternal A particle
KRAB-ZFP: Krüppel-associated box containing zinc finger protein
LTR: Long terminal repeat
PCR: Polymerase chain reaction
SNV: Single nucleotide variation
TEPBAT: Target Enrichment after Post-Bisulfite Adaptor Tagging
TF: Transcription factor
