# Supplementary material for: Locus-specific hypomethylation of the mouse IAP retrotransposon is associated with transcription factor-binding sites
Source: Mob DNA. 2017 Dec 13;8:20. doi: 10.1186/s13100-017-0105-0 (PMC5729234; doi:10.1186/s13100-017-0105-0)
Supplement: Supplementary file 1 — Sequence divergence of IAPEY_LTR genomic copies. (PDF 106 kb) [file 13100_2017_105_MOESM1_ESM.pdf]

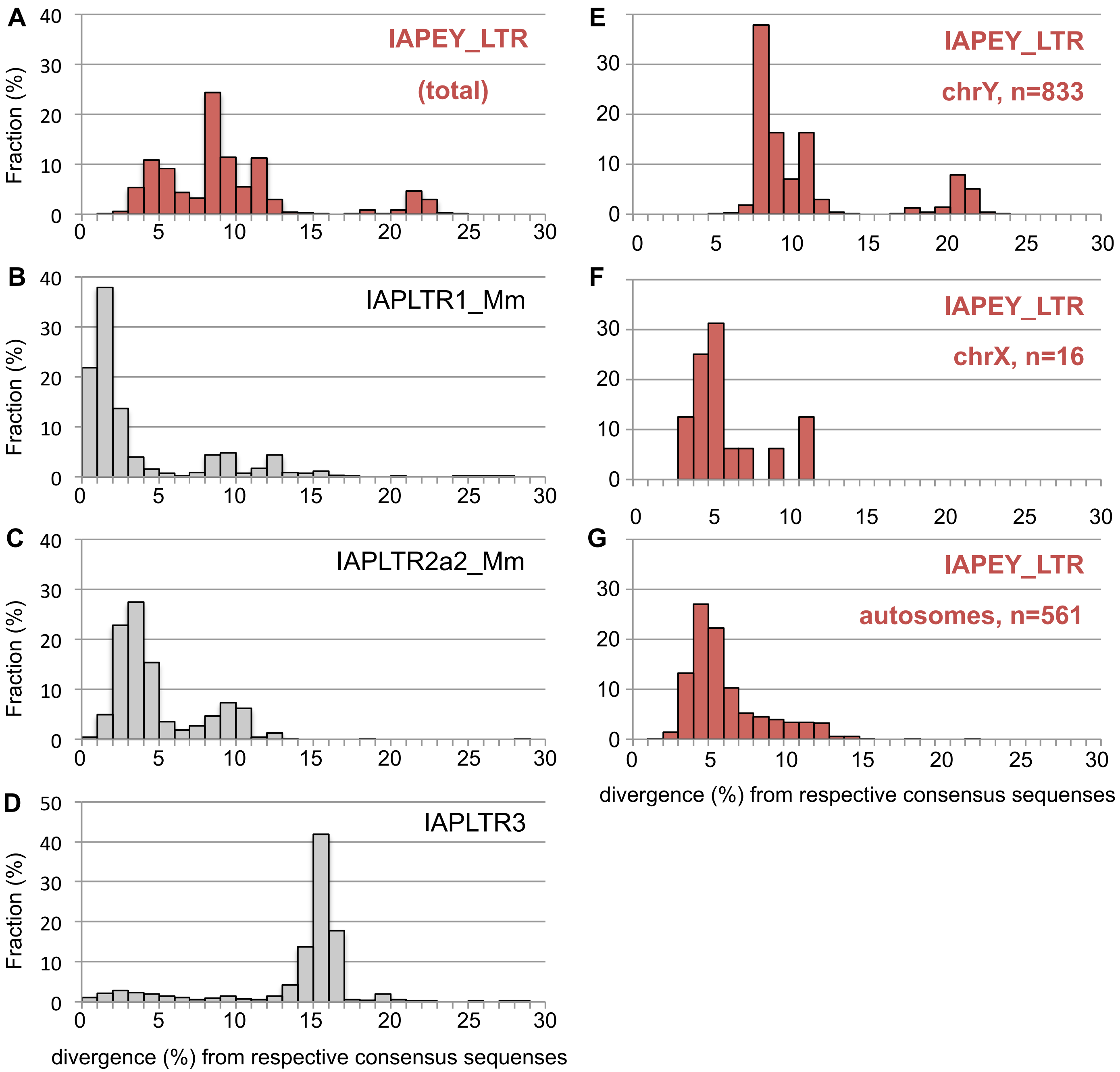

Figure S1. Sequence divergence of IAPEY_LTR genomic copies.
Sequence divergence (%) from the respective consensus sequences for genomic copies of IAPEY_LTR (A), IAPLTR1_Mm (B), IAPLTR2a2_Mm (c), and IAPLTR3 (D) are shown. For IAPEY_LTR sequences, statistics were analyzed separately for Y-chromosomal (E), X-chromosomal (F), and autosomal (G) copies. Although IAPEY copies have been accumulated in the Y chromosome, these copies are more divergent from the consensus sequence than those in autosomes.
